# Supplementary figures and images for: High-throughput nanopore sequencing of Treponema pallidum tandem repeat genes arp and tp0470 reveals clade-specific patterns and recapitulates global whole genome phylogeny
Source: Front Microbiol. 2022 Sep 20;13:1007056. doi: 10.3389/fmicb.2022.1007056 (PMC9531955; doi:10.3389/fmicb.2022.1007056)

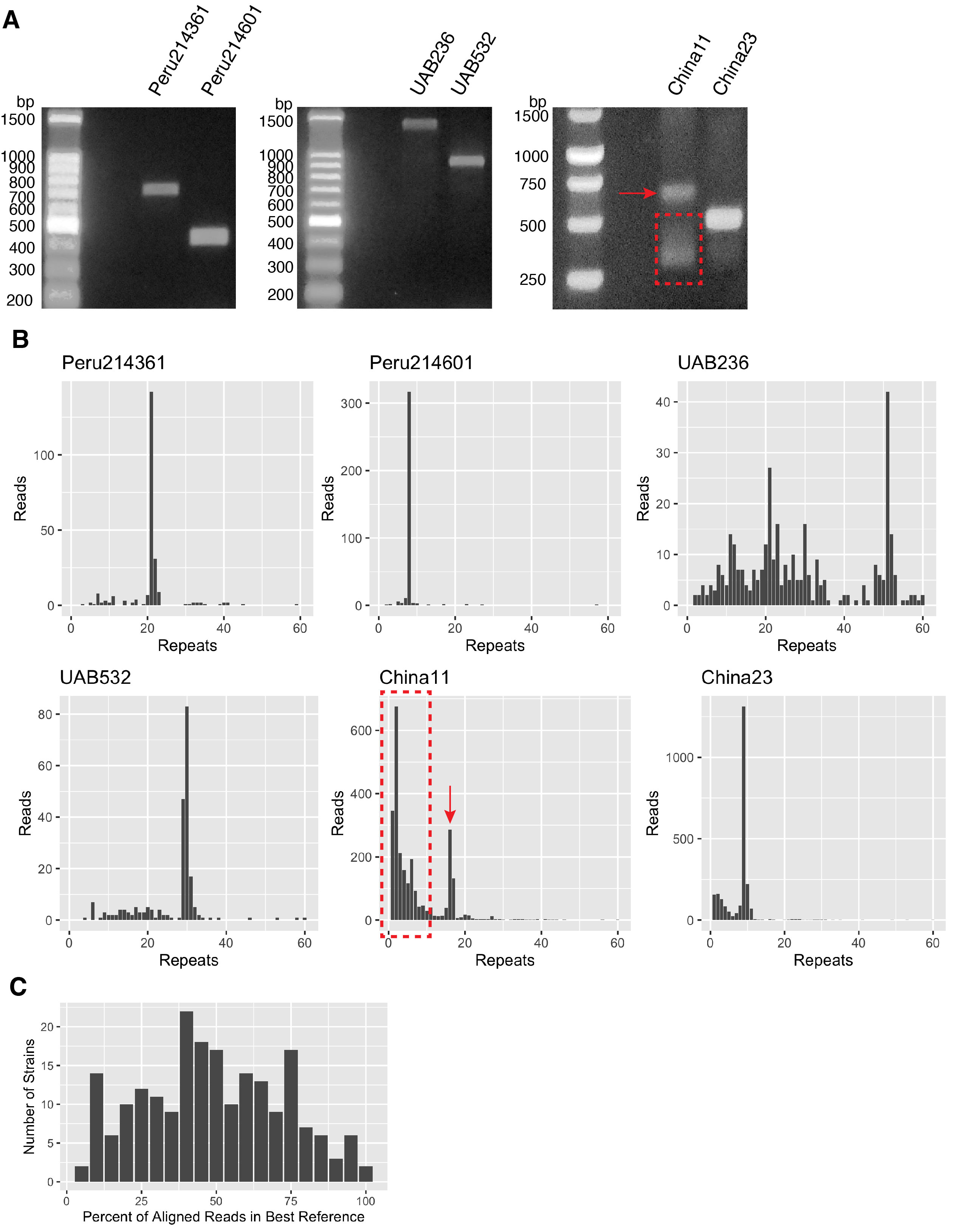

Supplement: Supplementary Figure 1 — tp0470 PCR band and nanopore pipeline call concordance of select samples. (A) PCR bands following barcoding. Bands should have a size of 24 bp times the number of repeats plus 273, including both flanking regions (225 bp) and two barcodes (48 bp total). The red box highlights non-specific amplification of low molecular weight fragments, while the red arrow shows the correct band size. (B) Histogram of the distribution of reads aligned to each length variant in the mapping reference file. (C) Distribution of the percent of all aligned reads in the top hit for all samples. [file Image_1.JPEG]

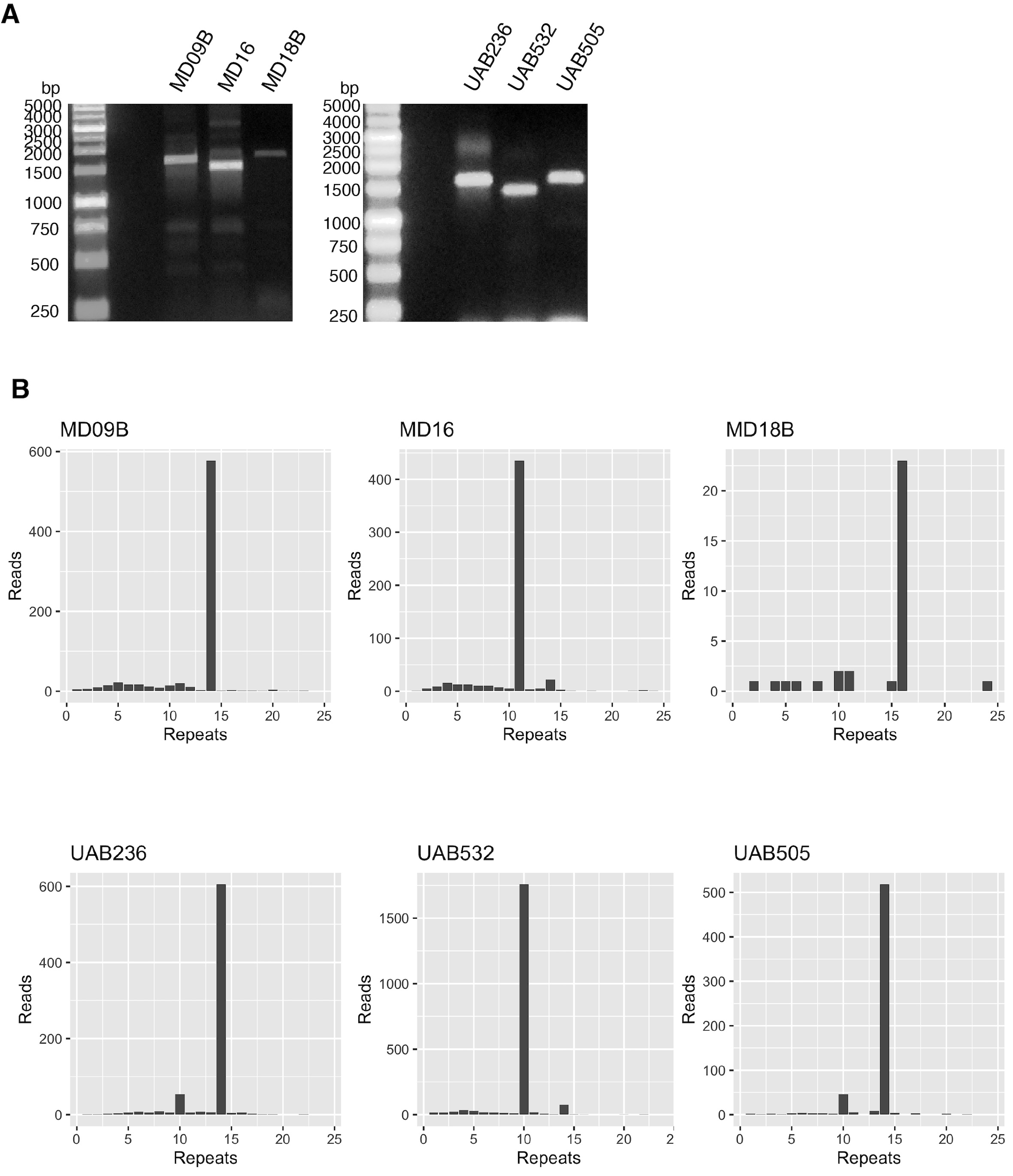

Supplement: Supplementary Figure 2 — arp PCR band and nanopore pipeline call concordance of select samples. (A) PCR bands following barcoding. Bands should have a size of 60 bp times the number of repeats plus 874, including both flanking regions (826 bp) and two barcodes (48 bp total). (B) Histogram of the distribution of reads aligned to each length variant in the mapping reference file. [file Image_2.JPEG]

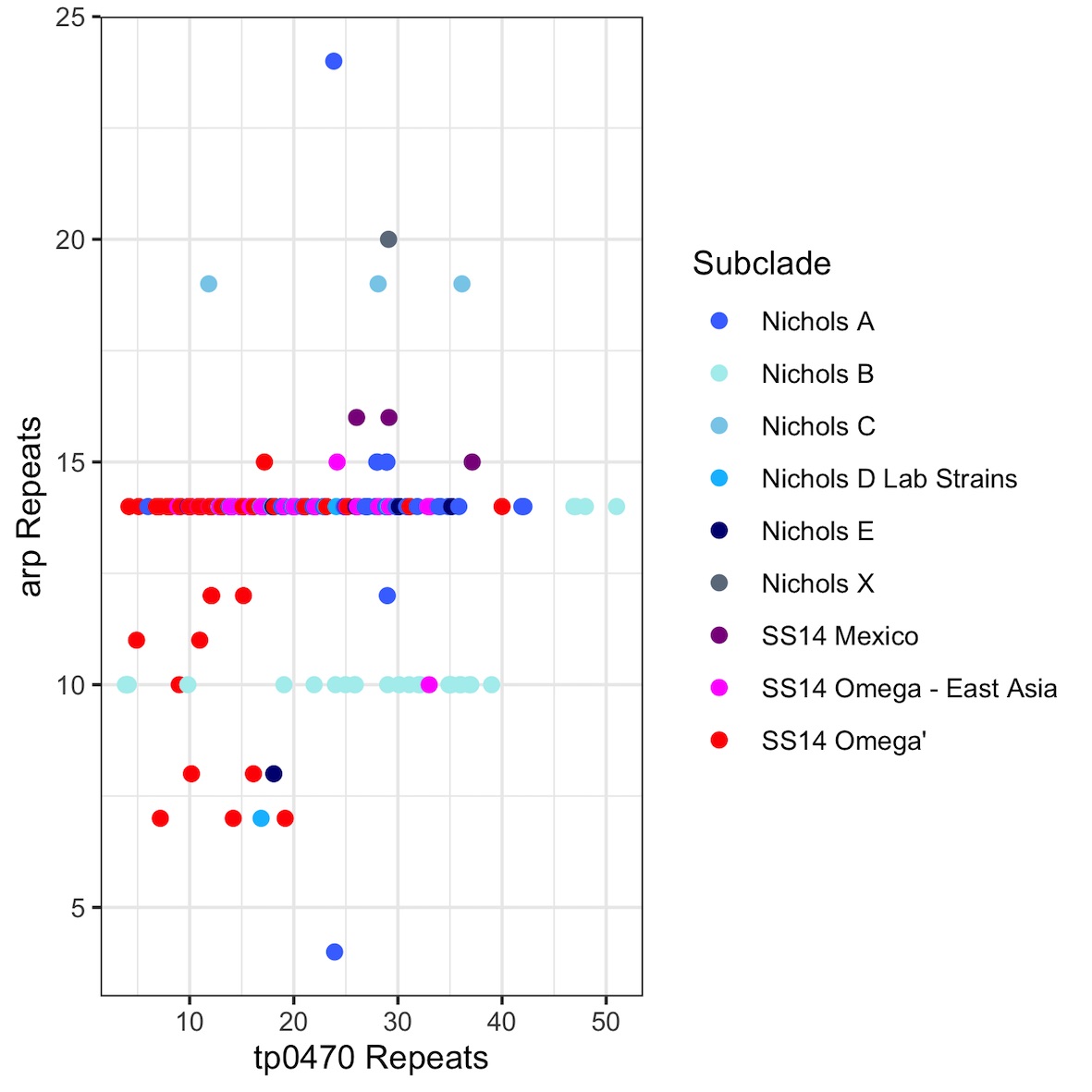

Supplement: Supplementary Figure 3 — arp repeat length is not correlated with tp0470 repeats. No correlation was seen between number of arp and tp0470 repeats (Pearson coefficient = 0.008). [file Image_3.JPEG]

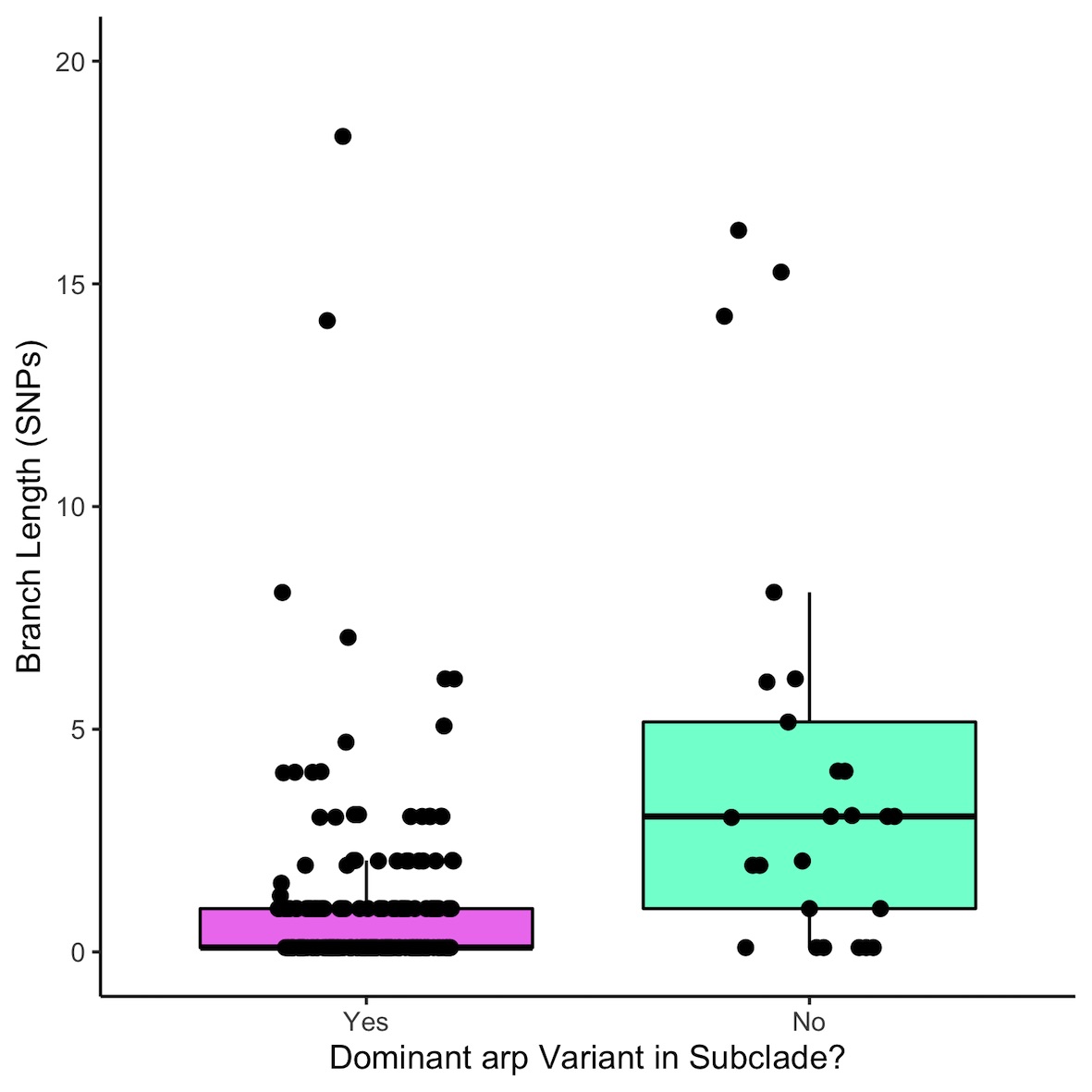

Supplement: Supplementary Figure 4 — Terminal branch lengths are longer for strains with non-dominant arp sequences. Average branch lengths from tip to ancestral node were determined for strains with dominant and non-dominant arp variants. p = 0.0046, Welch’s t-test. [file Image_4.JPEG]

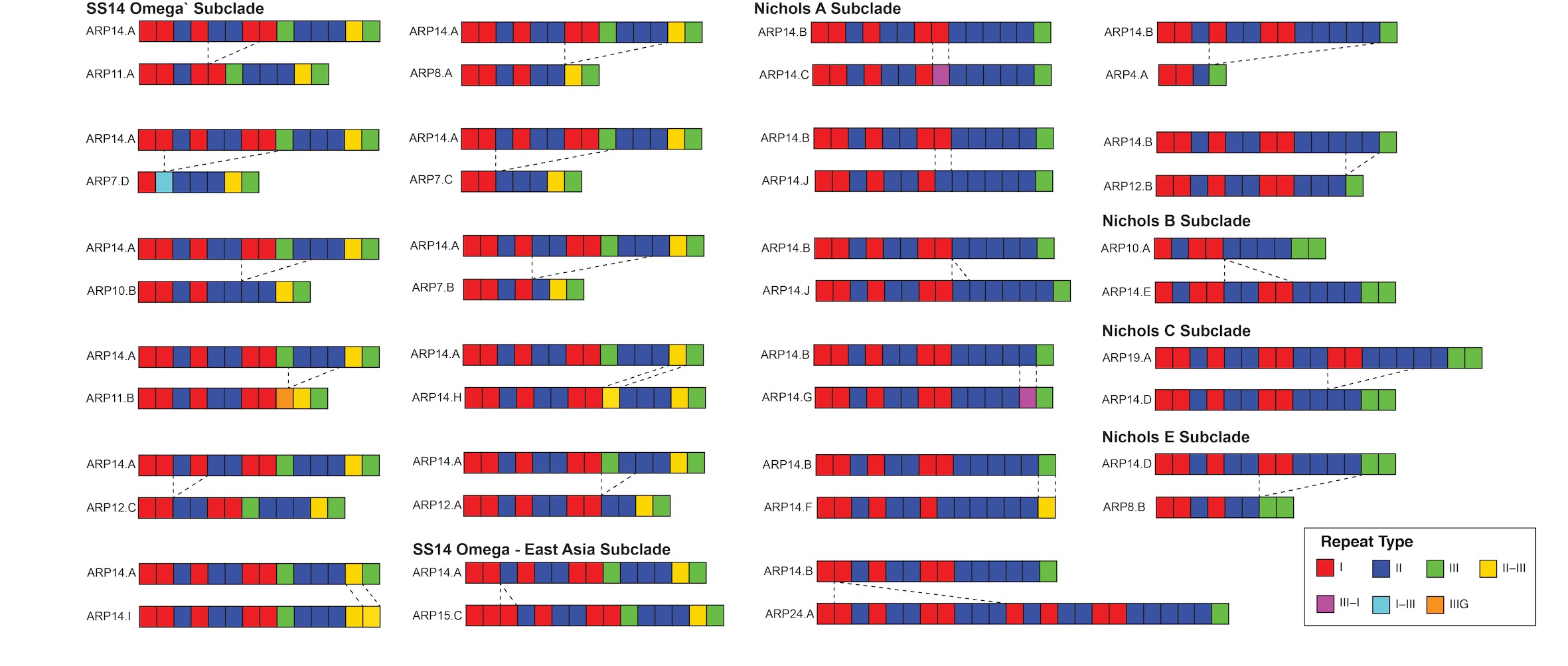

Supplement: Supplementary Figure 5 — Possible single recombination events to generate arp variants from the dominant arp variant in each subclade. The dominant sequence from each subclade is shown in the top position for each pair, and the non-dominant (possibly nascent) variant below. Dotted lines show possible junctions. Only a single possibility per variant is shown. [file Image_5.JPEG]

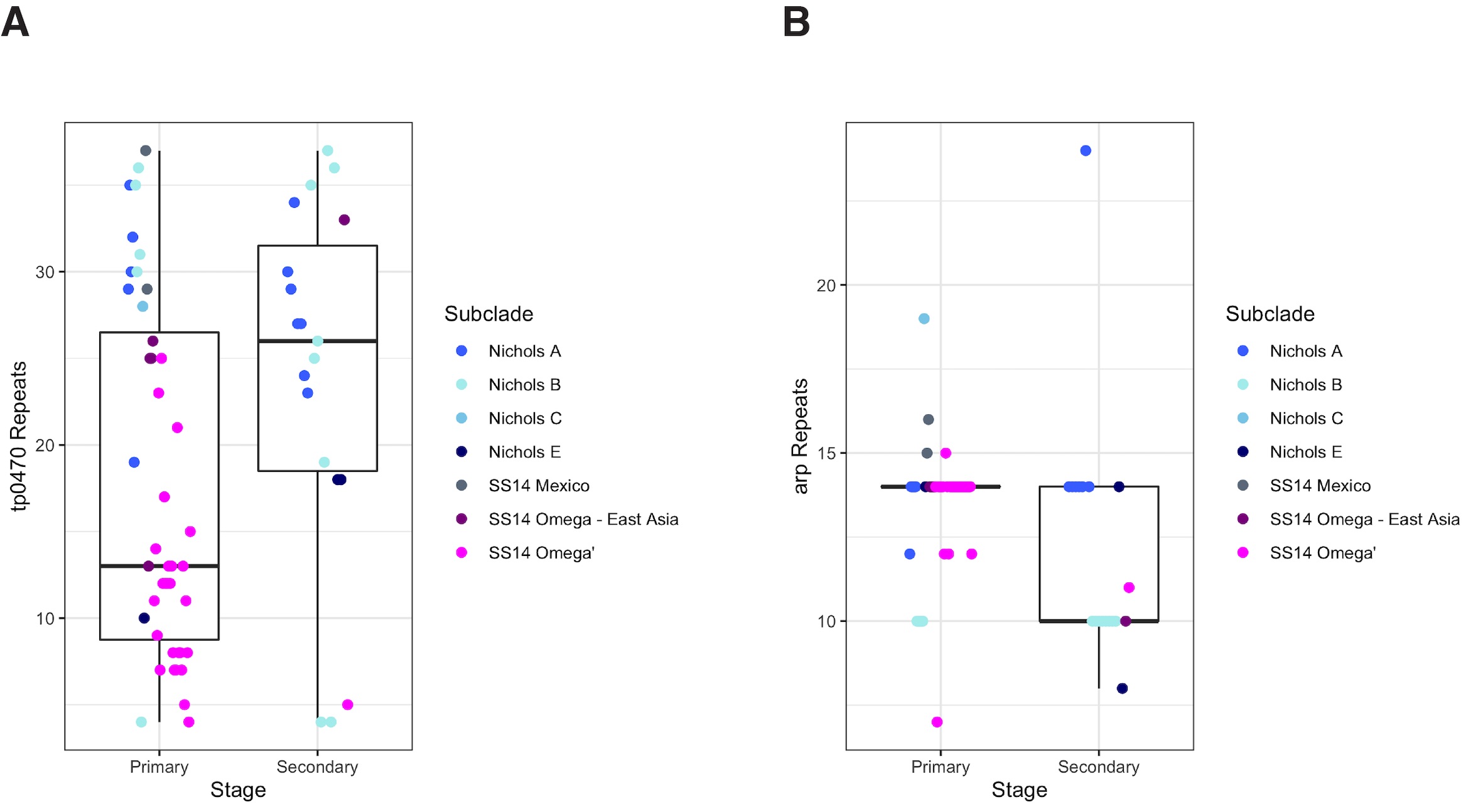

Supplement: Supplementary Figure 6 — Longer tp0470 repeats are associated with secondary syphilis. Out of 79 samples with stage information, samples from secondary syphilis had on average approximately five more repeats than primary p = 0.03624, Welch’s t-test. [file Image_6.JPEG]

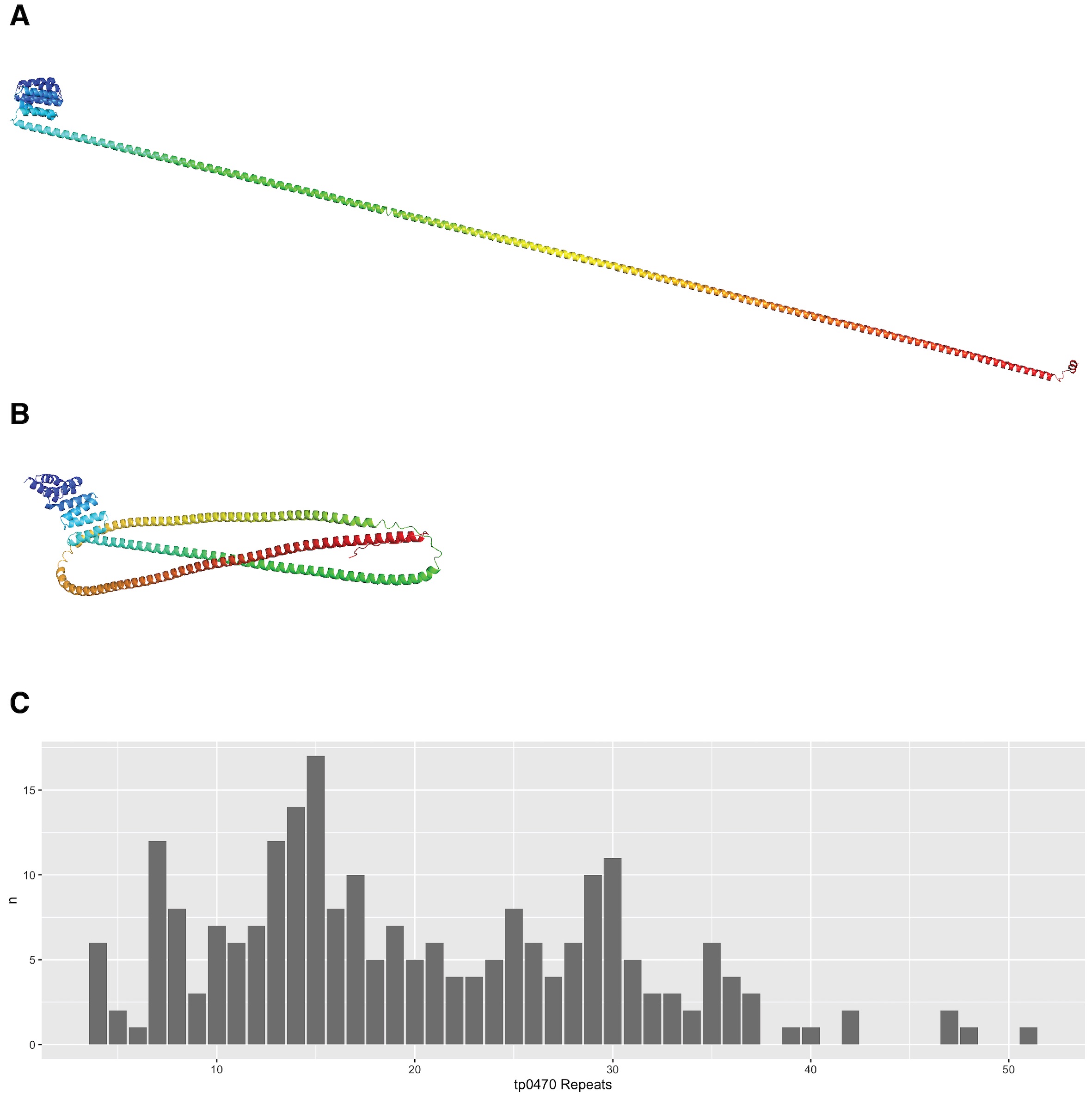

Supplement: Supplementary Figure 7 — TP0470 predictions: (A) trRosetta and (B) AlphaFold predictions of structure of 51 repeat TP0470 variant. Structures are colored from blue to red N term to C term. (C) Distribution of tp0470 variants in phylogeny. [file Image_7.JPEG]

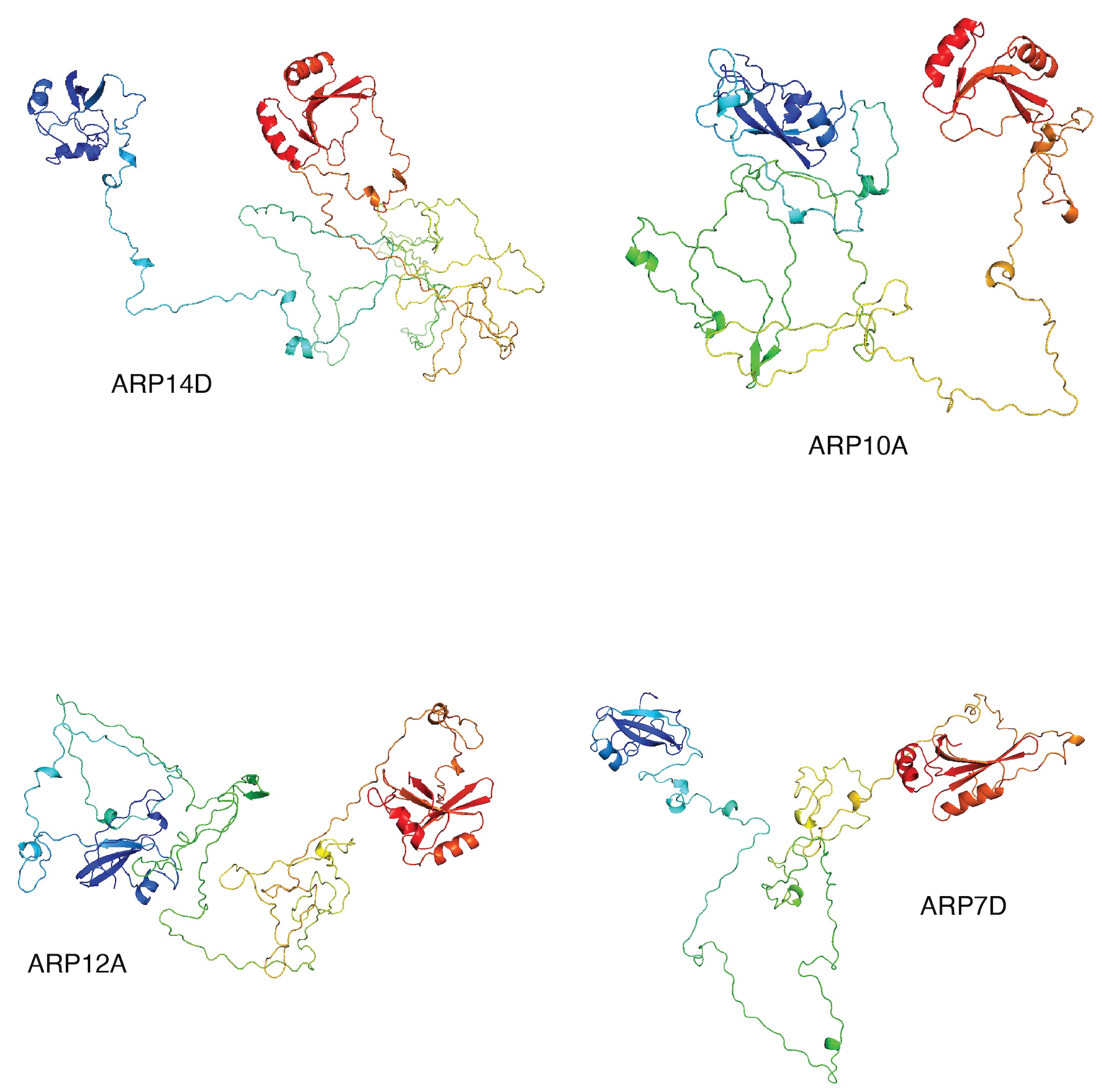

Supplement: Supplementary Figure 8 — trRosetta predictions for additional ARP variants. Structures are colored from blue to red N term to C term. [file Image_8.JPEG]
